# Sex differences of the association between handgrip strength and health-related quality of life among patients with cancer

**DOI:** 10.1038/s41598-024-60710-6

**Published:** 2024-04-30

**Authors:** Jihye Kim, Yujin Kim, Jae Won Oh, San Lee

**Affiliations:** 1 Department of Psychiatry, Yongin Severance Hospital, Yonsei University College of Medicine, Yongin, Republic of Korea; 2 Department of Social Welfare, Seoul National University, Seoul, Republic of Korea; 3 Department of Psychology, University of Utah Asia Campus, Incheon, Republic of Korea; 4 Department of Psychiatry and Institute of Behavioral Science in Medicine, Yonsei University College of Medicine, 50-1, Yonsei-ro, Seodaemun-gu, Seoul, Republic of Korea

**Keywords:** Handgrip strength, Health-related quality of life, KNHANES, Patients with cancer, Mental health, Population screening, Quality of life, Cancer, Health care, Medical research

## Abstract

The purpose of this study is to investigate the association between handgrip strength (HGS) and health-related quality of life (HRQoL), demonstrating HGS as an effective indicator for evaluating HRQoL of patients with cancer. Analyzing 1657 Korean adult cancer patients (644 males, 1013 females) aged ≥ 20 years from the Korea National Health and Nutrition Examination Survey (2014–2019), HGS was standardized based on body mass index and categorized by sex. HRQoL was assessed using the Euro Quality of Life-5-Dimension 3-Level version (EQ-5D-3L) Index. Lower relative HGS was associated with decreased HRQoL in female patients, while no significant association was found in male patients. The lowest quartile of relative HGS exhibited a 2.5-fold decrease in HRQoL compared to the highest quartile (OR 2.50, 95% CI 1.59–3.95, *p* < 0.001). Both male and female patients with cancer were affected by age, subjective health perception, and stress recognition regarding HRQoL. This study suggests that HGS may be associated with the HRQoL of female patients with cancer, emphasizing that the HGS measurement can be effectively utilized as a pivotal tool for evaluating HRQoL in female patients with cancer.

As of 2020, cancer continues to be the leading cause of death in South Korea, which has a population of nearly 247,952 patients with cancer. The number of cancer survivors (those diagnosed after 1999 and still in treatment or considered cured as of January 1, 2021) reached approximately 2.28 million in 2020, showing an increase of approximately 130,000 compared with the previous year^1^. When comparing 5-year survival rates for selected cancer types covered by the National Cancer Screening Program internationally, South Korea generally exhibits higher survival rates than countries like the United States and the United Kingdom^2^. The 5-year survival rate for patients with cancer who were diagnosed in the most recent 5 years (2016–2020) is 71.5%, indicating that 7 out of 10 patients with cancer survive for at least 5 years. This rate has been steadily increasing since 1993, with a notable increase of 6.0% from the rate of 65.5% observed in the 2006–2010 period. The probability of developing cancer in South Korea is estimated to be 36.9% if citizens survive up to their life expectancy (83.5 years), with a 39.0% likelihood for men (life expectancy of 80.5 years) and a 33.9% likelihood for women (life expectancy of 86.5 years)^3^.

Health-related quality of life (HRQoL) is crucial in cancer treatment and recovery. This is a subjective and complex measure encompassing physical, psychological, and social aspects^4^. Patients with cancer experience various challenges during their treatment journey, including treatment side effects, physical symptoms, job and role responsibilities, all of which should be assessed to ensure their well-being^5^. During and after completion of the treatment process, patients also experience various side effects and discomfort^6^. Moreover, along with the physical pain experienced due to side effects, patients also experience social and psychological issues, including fear of prognosis and death, anxiety, depression, isolation, and despair^7^. Long-term challenges resulting from treatment, emotional distress, treatment costs, economic hardships due to job loss, and family conflicts contribute to the difficulties in psychological and social adaptation. These difficulties may further lead to mental health issues like depression and stress, which directly or indirectly impact the HRQoL of patients with cancer^8^.

Exercise and physical activity have been reported to positively impact the physical and psychological health of patients with cancer, in addition to improving their overall HRQoL^9^. Handgrip strength (HGS) is the force exerted by the muscles when holding an object using all four fingers and the thumb^10^, and HGS is known as a relatively simple and cost-effective method to measure muscular strength^11^. HGS has been associated with reduced mortality rates^12^, with a reported 4% reduction in mortality per 1-kg increase in HGS. Correspondingly, lower HGS has been linked to a 79% higher mortality rate^13^. Decreased muscular strength is associated with various disabilities and diseases among patients with cancer, contributing to multiple health issues and affecting mortality^14^. Some studies emphasize the positive effects and importance of increasing muscular strength in patients with cancer^15,16^. Therefore, the HGS of patients with cancer should be assessed and the relationship between HGS levels and HRQoL should be understood, in order to apply psychological intervention strategies to attempt to improve the HRQoL of patients with cancer.

While previous studies have reported the positive effects and significance of strength training for patients with cancer^17–19^, there is scarce research on the impact of HGS specifically as an effect of strength training on these patients. A study conducted in Canada indirectly demonstrates the importance of strength training for patients with cancer^20^. In this study, patients with breast cancer undergoing cancer treatment were divided into groups receiving either strength training combined with aerobic exercise or aerobic exercise alone. The results showed that the group receiving both strength training and cancer treatment experienced significantly improved cancer-related symptoms compared with the group receiving only aerobic exercise. Furthermore, in a study by An, Kang, Min^21^ which is unique in Korea for examining the relationship between HGS and HRQoL in patients with cancer using data from the Korea National Health and Nutrition Examination Survey, patients with cancer were found to show lower relative HGS, as well as significantly lower HRQoL indices, compared with the higher HGS group.

Research on the effects of strength training on patients with cancer is still limited, and the relationship between strength and the HRQoL of patients with cancer has not been extensively explored. Therefore, this study aims to investigate the influence of HGS on the HRQoL of adult patients with cancer using data from the Korean National Health and Nutrition Examination Survey (KNHANES) conducted from 2014 to 2018. The purpose of this study was to utilize HGS as an indicator of muscular strength in patients with cancer, examining its association with HRQoL. Through this, we aimed to explore the utility of HGS as an indirect metric reflecting HRQoL.

## Methods

### Study design and participants

The KNHANES is a comprehensive national survey aimed at gauging the health and nutritional condition of Koreans. Providing extensive data like the United States' National Health and Nutrition Examination Survey, it offers vital statistics from the general population of Korea. Its primary goal is to assess the health and nutritional state of South Koreans and to supply crucial information for the creation and assessment of health policies and initiatives within the nation. The KNHANES is conducted annually in 192 regions of Korea under the supervision of the Korea Disease Control and Prevention Agency, and includes medical examinations, health surveys, and nutrition surveys. Detailed information on the monitoring process is available to the public and can be obtained from the official KNHANES website (http://knhanes.kdca.go.kr)^22^.

**Figure 1:**
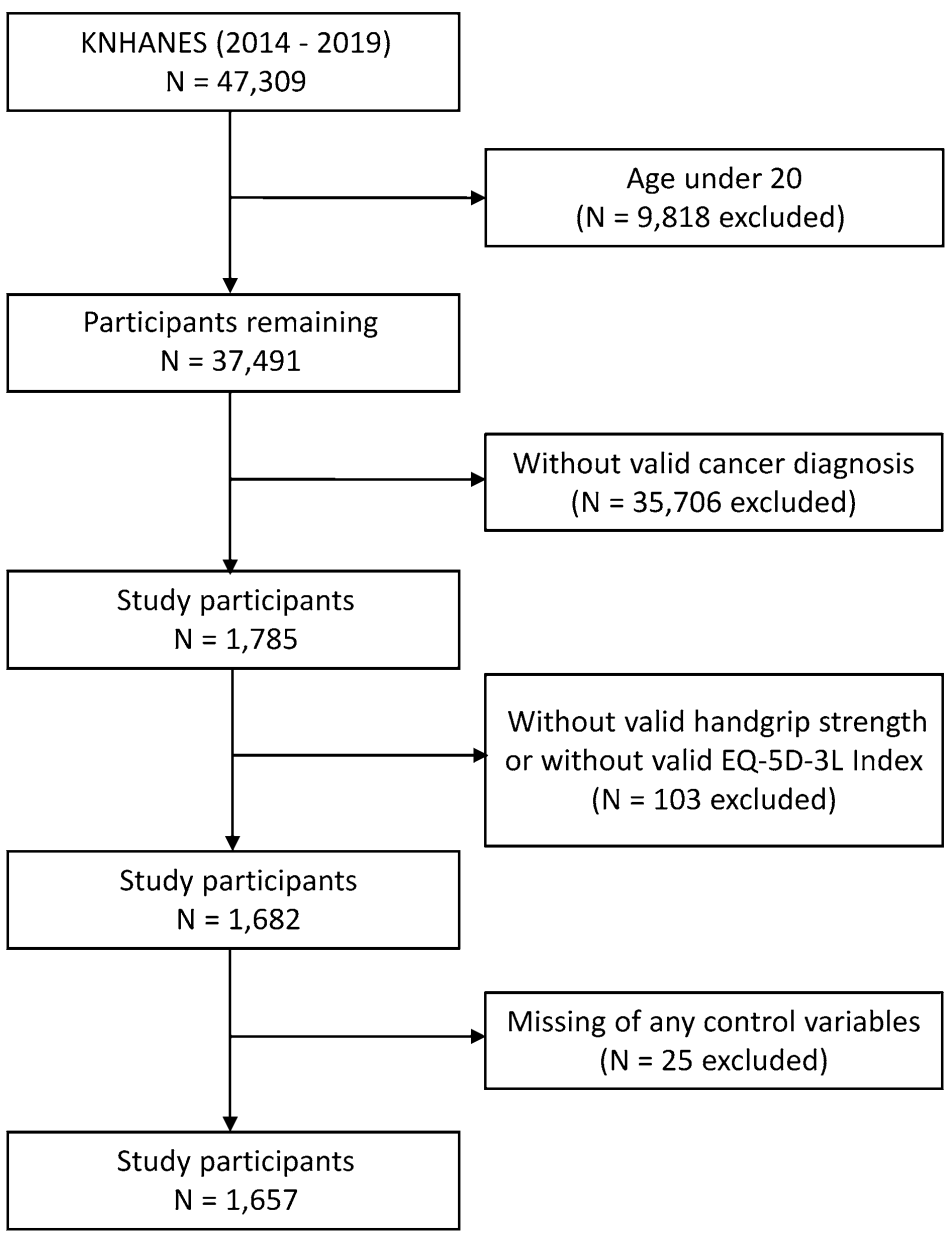
Flow diagram of the study participants. KNHAES, Korea National Health and Nutritional Examination Survey, EQ-5D-3L index, Euro Quality of Life-5-Dimension 3-Level index.

This study was conducted through secondary analysis using raw data provided by the 6th NHANES from 2014 to 2015, the 7th NHANES from 2016 to 2018, and the 8th NHANES in 2019. From the extensive data of the KNHANES, relevant variables for precursor studies and related research on cancer were extracted to analyze the relationship between HGS and HRQoL in adult patients with cancer ≥ aged 20 years. Over the six-year period from 2014 to 2019, out of 47,309 respondents, a total of 37,491 individuals aged 20 years and older met the inclusion criteria. After excluding individuals with incomplete data among cancer patients, a total of 1657 individuals (644 men and 1013 women) were included in the study based on the main variables without missing data. (Fig. 1). In South Korea, the total prevalent cancer cases in 2019 were 2,147,503. It suggested that 4.2% of the entire Korean population had a history of being diagnosed with cancer in 2019^23^. In comparison, the participants aged 20 and over from the KNHANES between 2014 and 2019 used in this study numbered 37,491, of which 1657 were patients with cancer, accounting for 4.4%. By excluding individuals under 20, who have a relatively lower prevalence of cancer, the proportion of patients with cancer in this study shows a slight increase compared to national statistics. All participants signed an informed consent form, and the KNHANES is conducted in accordance with the ethical standards of the Declaration of Helsinki. The KNHANES database provides publicly available, de-identified secondary data. This study received exemption from review approval from the Institutional Review Board of Yongin Severance Hospital (Approval No. 9–2023-0179).

### Measures

#### Diagnosis and related variables of cancer 

A patient with cancer was defined as a patient who had been diagnosed with any type of cancer. The following types of cancer were included: stomach cancer, liver cancer, colorectal cancer, breast cancer, cervical cancer, lung cancer, thyroid cancer, and other cancers. The definition of “patients with cancer” was confirmed through the KNHANES. Among the health survey questions, participants who responded “yes” to the item regarding the “diagnosis by a doctor” for cancers listed above were classified as “patients with cancer.”

#### Handgrip strength

HGS was measured using a digital grip strength dynamometer (Digital grip strength dynamometer, TKK-5401, Takei, Japan) in the KNHANES. The maximum value among the 1st, 2nd, and 3rd HGS of the dominant hand was defined as the HGS value. High correlation with anthropometric measurements, such as weight and height, were observed, and previous studies also used the relative HGS value obtained by dividing HGS by body mass index (BMI)^23^. Therefore, the relative HGS value divided by BMI (kg/m^2^) was used as the final HGS value in this study^24^. Relative HGS was analyzed by dividing it into quartiles (Q1, Q2, Q3, Q4) separately for each sex, and the category with the highest relative HGS was set as Quartile 1.

#### Health-related quality of life

HRQoL was measured using the EQ-5D-3L scale developed by the EuroQoL Group. This measurement tool includes five dimensions: mobility, self-care, usual activities, pain/discomfort, and anxiety/depression. Respondents indicated their level of impairment in each dimension using three categories (no problem, some problems, extreme problems). The QoL index score was derived using quality weights estimated by the Korea Disease Control and Prevention Agency, resulting in a score ranging from − 0.171 to 1. A higher score indicates a better QoL. The participants with EQ-5D-3L index scores were divided into the high or low QoL groups based on the threshold value for each sex. Therefore, scores that were lower than the threshold were considered to indicate low HRQoL, while scores equal to or higher than the threshold indicated high HRQoL. The EQ-5D-3L Index was analyzed by dividing it into two categories (low, high) separately for each sex.

### Covariates

Sociodemographic characteristics of the participants, including sex, age, educational levels, household income levels, and marital status, were included. Sex was classified as “male” and “female,” while age was categorized as “under 65” and “65 or above.” Educational levels were categorized as “elementary school or below,” “middle school graduation,” “high school graduation,” and “university graduation or higher.” Household income levels were divided into four quartiles: upper, upper-middle, lower-middle, and lower. Marital status was categorized as married and not married (single, death, divorce, or separation).

The health-related characteristics of the participants included smoking status, alcohol consumption, and subjective health status. Based on smoking status, patients were classified into “smoker” for those who are currently smoking and “non-smoker” for the rest. All patients who consumed alcohol were reclassified as “drinkers,” excluding those who responded, “did not drink at all in the past year.” Subjective health status was measured through a question about one's overall health perception. The question asked was, “How do you perceive your overall health status?” with responses on a 5-point Likert scale ranging from ‘very good’, ‘good’, ‘fair’, ‘bad’, to ‘very bad’. Higher scores indicate a better perceived subjective health status. In our study, we combined ‘very good’ and ‘good’ into a single ‘good’ group, and ‘bad’ and ‘very bad’ into a ‘bad’ group, thus analyzing the data in three groups: ‘good’, ‘fair’, and ‘bad’. The psychological well-being characteristics included the recognition of stress. “Feeling a lot” and “feeling quite a bit” were classified as “yes,” while “feeling a little” and “almost never feeling” were categorized as “no”.

### Statistical analysis

The statistical analysis in this study was conducted using SAS 9.4 (SAS Institute Inc., Cary, NC, USA). The demographic characteristics and distribution of each variable according to the HRQoL of the male and female patients with cancer were presented as numbers and percentages and compared using the chi-square test. In addition, multivariable logistic regression analysis was performed to investigate the association between relative HGS and HRQoL. In the analysis, the high HRQoL group was set as the reference, to determine whether the odds ratio (OR) for low HRQoL was significantly different. ORs and 95% confidence intervals (CIs) were calculated, with statistical significance considered as *p* < 0.05. The “metafor” package in R (version 4.3.1) was used to visualize the data into forest plots.

## Results

### General characteristics

This study included a total of 1657 adults aged ≥ 20 years, with a mean age of 51.6 (standard deviation 16.5) years. Among them, 644 were male (39.9%) and 1013 were female (61.1%). The baseline characteristics of the study participants are presented in Table 1. Due to the number of participants that matched the median value of HRQoL exactly, the two groups divided by HRQoL in each sex were not evenly divided. In this study, we categorized males and females into two groups based on Euro Quality of Life-5-Dimension 3-Level version (EQ-5D-3L) Index values to distinguish between low and high quality of life using a threshold^25^. In the male group, the mean EQ-5D-3L index was 0.93, with a minimum value of 0 and a maximum value of 1. For the female group, the mean EQ-5D-3L index was 0.92, with a minimum value of 0.08 and a maximum value of 1. This result indicates that the distribution of the data is skewed towards the maximum value of the EQ-5D-3L. In the male group, the EQ-5D-3L index was distributed as follows: 226 (35.1%) in the low group and 418 (64.9%) in the high group. In the female group, the distribution was 432 (42.7%) in the low group and 581 (57.3%) in the high group.

**Table 1:** Study characteristics according to sex and health-related quality of life in patients with cancer.

Variables	Male (N = 644; 38.9%)			Female (N = 1013; 61.1%)		
	EQ-5D-3L index		*P* value	EQ-5D-3L index		*P* value
	Low	High		Low	High	
HGS/BMI ratio (kg/BMI)			**0.002**			** < 0.001**
Quartile 1 (high)	49 (30.4)	112 (69.6)		62 (24.5)	191 (75.5)	
Quartile 2	45 (27.9)	116 (72.1)		101 (39.8)	153 (60.2)	
Quartile 3	53 (32.9)	108 (67.1)		110 (43.5)	143 (56.5)	
Quartile 4 (low)	79 (49.1)	82 (50.9)		159 (62.9)	94 (37.1)	
Age			** < 0.001**			** < 0.001**
Under 65	58 (24.4)	180 (75.6)		208 (33.1)	421 (66.9)	
65 or more	168 (41.4)	238 (58.6**)**		224 (58.3)	160 (41.7)	
Educational attainment			**0.005**			** < 0.001**
Elementary school and below	82 (44.6)	102 (55.4)		206 (60.2)	136 (39.8)	
Middle school	38 (36.9)	65 (63.1)		55 (42.6)	74 (57.4)	
High school	54 (32.5)	112 (67.5)		107 (34.3)	205 (65.7)	
University or above	52 (27.2)	139 (72.8)		64 (27.8)	166 (72.2)	
Equalized household income			** < 0.001**			** < 0.001**
Quartile 1 (high)	28 (20.0)	112 (80.0)		68 (29.7)	161 (70.3)	
Quartile 2	33 (25.0)	99 (75.0)		103 (38.6)	164 (61.4)	
Quartile 3	72 (36.5)	125 (63.5)		104 (40.5)	153 (59.5)	
Quartile 4 (low)	93 (53.1)	82 (46.9)		157 (60.4)	103 (39.6)	
Marital status			**0.001**			** < 0.001**
Married	179 (32.2)	377 (67.8)		271 (37.4)	453 (62.6)	
Not married	47 (53.4)	41 (46.6)		161 (55.7)	128 (44.3)	
Alcohol use status			**0.044**			**0.035**
No	121 (39.0)	189 (61.0)		334 (44.6)	415 (55.4)	
Yes	105 (31.4)	229 (68.6)		98 (37.1)	166 (62.9)	
Smoking status			0.956			0.374
Non-smoker	195 (35.1)	360 (64.9)		422 (42.5)	572 (57.6)	
Smoker	31 (34.8)	58 (65.2)		10 (52.6)	9 (47.4)	
Subjective health status			** < 0.001**			** < 0.001**
Good	24 (15.5)	131 (84.5)		32 (18.4)	142 (81.6)	
Fair	94 (31.3)	206 (68.7)		172 (34.7)	324 (65.3)	
Bad	108 (57.1)	81 (42.9)		228 (66.5)	115 (33.5)	
Stress recognition			**0.003**			** < 0.001**
No	175 (32.6)	362 (67.4)		294 (38.1)	478 (61.9)	
Yes	51 (48.7)	56 (52.3)		138 (57.3)	103 (42.7)	
*Total*	226 (35.1)	418 (64.9)		432 (42.7)	581 (57.3)	

*EQ-5D-3L index* Euro Quality of Life-5-Dimension 3-level version index, *HGS/BMI ratio* handgrip strength/BMI ratio. Significant values are in bold, indicating significance at *p* < 0.05, *p* < 0.01, or *p* < 0.001 levels.

Regarding HGS, statistically significant HRQoL differences were observed for both men and women (*P* = 0.002 and < 0.001, respectively), whereby men aged ≥ 65 years (58.6%, *P* < 0.001) and women aged ≥ 65 years (41.7%, *P* < 0.001) showed lower HRQoL. Educational levels correlated with higher QoL for those with a university education or higher in both men and women (*P* = 0.005 and < 0.001, respectively). In terms of household income levels, higher income levels were associated with higher HRQoL in both sexes (both *P* < 0.001). Both married men (67.8%, *P* = 0.001) and women (62.6%, *P* < 0.001) demonstrated higher HRQoL. Being a current drinker showed a significant association with lower HRQoL in both men and women (*P* = 0.044 and 0.035, respectively), whereas being a current smoker did not demonstrate a significant relationship with HRQoL. Good subjective health status and no stress recognition were significantly associated with higher HRQoL in both sexes.

### Association between HGS and HRQoL

**Figure 2:**
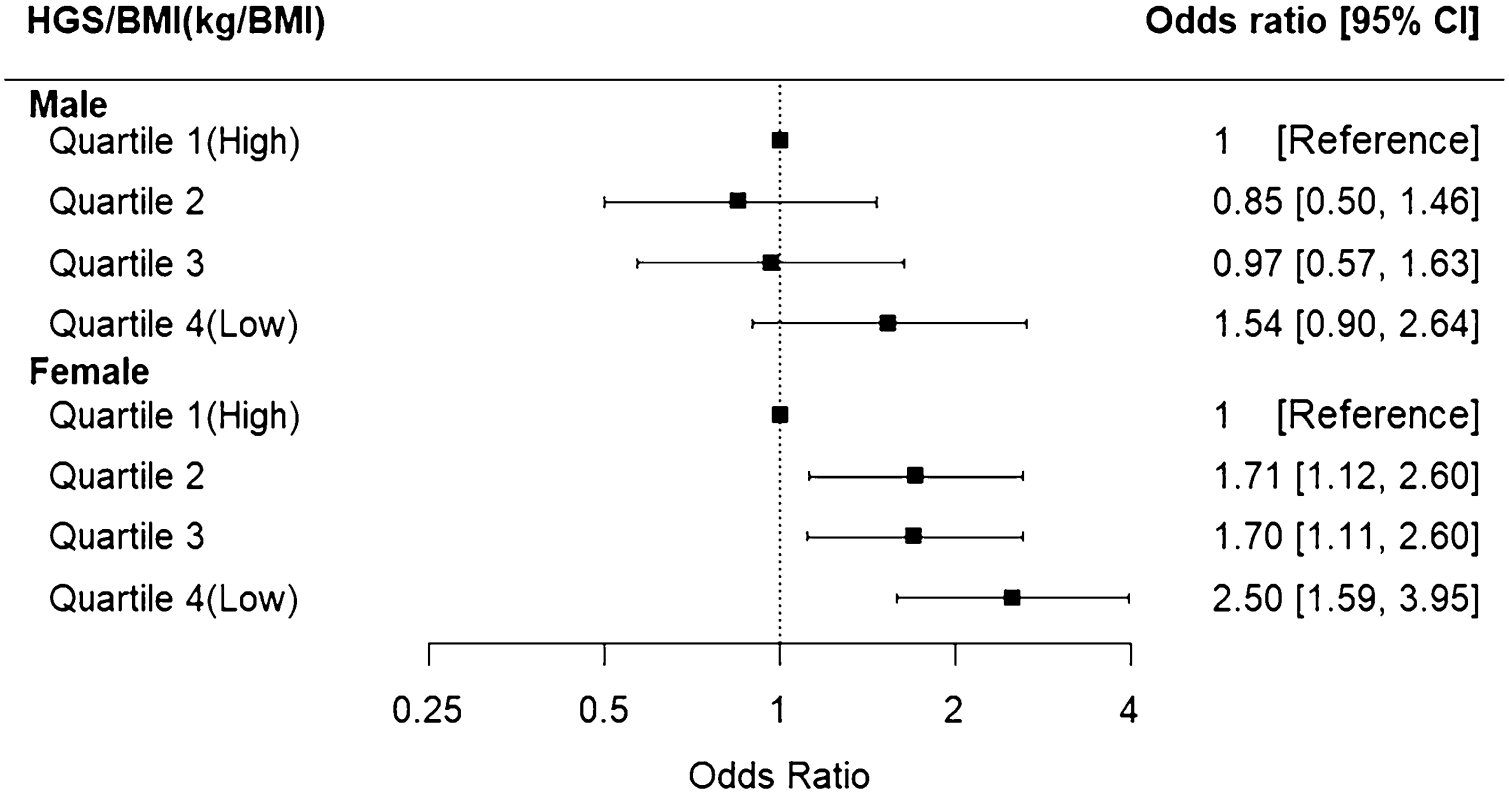
Forest plot of grip strength and the health-related quality of life in male and female patients with cancer. Adjusted for educational attainment level, equalized household income, marital status, alcohol use status, smoking status, subjective health status, and stress recognition category. EQ-5D-3L index, Euro Quality of Life-5-Dimension 3-Level index; HGS/BMI ratio, handgrip strength/body mass index ratio; CI, Confidence Interval.

The results of multivariable logistic regression analysis, demonstrating the relationship between HGS and HRQoL stratified by sex, are presented in Fig. 2. Quartile 1 was defined as high relative HGS and used as a reference to determine the association between HGS levels and HRQoL. For men, no significant differences were observed. However, for women, as HGS decreased, there was a trend of decreasing HRQoL in Quartile 2 (OR 1.71, 95% CI 1.12–2.60, *P* = 0.012), in Quartile 3 (OR 1.70, 95% CI 1.11–2.60, *P* = 0.014), and in Quartile 4 (OR 2.50, 95% CI 1.59–3.95, *P* < 0.001).

### Covariates and HRQoL

In the multivariable logistic regression, the associations between HRQoL and all covariates, except relative HGS, are presented in Table 2. For men, compared to those aged ≥ 65 years, patients aged < 65 years had lower odds of low HRQoL (OR 0.51, 95% CI 0.32–0.81, *P* = 0.005). Patients in the third income quartile showed lower HRQoL compared with those in the fourth (OR 2.25, 95% CI 1.23–4.15, *P* = 0.008). Similarly, patients who were not married showed lower HRQoL compared with those who were married (OR 1.93, 95% CI 1.15–3.24, *P* = 0.014). The perception of subjective health was associated with lower HRQoL in patients with “normal” (OR 2.33, 95% CI 1.38–3.39, *P* = 0.002) and “poor” (OR 5.88, 95% CI 3.32–10.24, *P* < 0.001) subjective health levels compared with “good” ones. Similarly, experiencing stress was associated with lower HRQoL (OR 2.04, 95% CI 1.25–3.34, *P* = 0.005).

**Table 2:** Results of multivariable logistic regression analysis of the association between covariates and health-related quality of life.

Variables	EQ-5D-3L index							
	Male				Female			
	OR	95% CI		*P* value	OR	95% CI		*P* value
HGS/BMI ratio(kg/BMI)								
Quartile 1 (high)	1.00				1.00			
Quartile 2	0.85	0.50	1.46	0.560	1.71	1.12	2.60	**0.012**
Quartile 3	0.97	0.57	1.63	0.908	1.70	1.11	2.60	**0.014**
Quartile 4 (low)	1.54	0.90	2.64	0.119	2.50	1.59	3.95	** < 0.001**
Age								
Under 65	**0.51**	**0.32**	**0.81**	**0.005**	**0.68**	**0.47**	**0.98**	**0.038**
65 or more	1.00				1.00			
Educational attainment								
Elementary school and below	1.00				1.00			
Middle school	0.75	0.44	1.28	0.294	**1.82**	**1.14**	**2.91**	**0.013**
High school	0.84	0.46	1.52	0.562	1.21	0.72	2.02	0.475
University or above	0.80	0.48	1.36	0.411	1.28	0.84	1.93	0.251
Equalized household income								
Quartile 1 (high)	1.06	0.57	1.97	0.851	1.31	0.86	2.01	0.211
Quartile 2	1.52	0.86	2.71	0.153	0.99	0.63	1.54	0.947
Quartile 3	**2.25**	**1.23**	**4.15**	**0.008**	1.23	0.75	2.01	0.407
Quartile 4 (low)	1.00				1.00			
Marital status								
Married	1.00				1.00			
Not married	**1.93**	**1.15**	**3.24**	**0.014**	1.41	1.00	1.98	0.048
Alcohol use status								
No	1.00				1.00			
Yes	1.00	0.69	1.45	0.989	1.02	0.73	1.41	0.922
Smoking status								
Non-smoker	1.00				1.00			
Smoker	1.12	0.65	1.91	0.701	1.37	0.50	3.77	0.544
Subjective health status								
Good	1.00				1.00			
Fair	**2.33**	**1.38**	**3.93**	**0.002**	**1.93**	**1.23**	**3.02**	**0.004**
Bad	**5.83**	**3.32**	**10.24**	** < 0.001**	**6.06**	**3.77**	**9.74**	** < 0.001**
Stress recognition								
No	1.00				1.00			
Yes	**2.04**	**1.25**	**3.34**	**0.005**	**1.68**	**1.20**	**2.35**	**0.002**

*EQ-5D-3L index* Euro Quality of Life-5-Dimension 3-Level index, *HGS/BMI ratio* handgrip strength/body mass index ratio, *OR* odds ratio, *CI* confidence interval. Significant values are in bold, indicating significance at *p* < 0.05, *p* < 0.01, or *p* < 0.001 levels.

For women, compared with those aged ≥ 65 years, patients < 65 years had lower HRQoL (OR 0.68, 95% CI 0.47–0.98, *P* = 0.038). Patients with intermediate education had lower HRQoL compared to those with primary education (OR 1.82, 95% CI 1.14–2.91, *P* = 0.013). Perception of subjective health and stress recognition showed similar trends for men, with those experiencing “normal” (OR 1.93, 95% CI 1.23–3.02, *P* = 0.004) and “poor” (OR 6.06, 95% CI 3.77–9.74, *P* < 0.001) levels showing lower HRQoL than those experiencing “good” subjective health levels. Moreover, stress recognition was associated with lower HRQoL (OR 1.68, 95% CI 1.20–2.35, *P* = 0.002).

## Discussion

The results of this study revealed a significant association between HGS and HRQoL in female patients with cancer. HRQoL was found to decrease with relative decrease in HGS. However, no statistically significant association between relative HGS and HRQoL was observed among male patients with cancer.

The quality of life for female patients with cancer has been shown to be affected by the relationship between functional status, psychological distress (anxiety, depression), and the degree of QoL. As the functional status score increases, the ability to perform activities decreases, psychological distress increases, and QoL decreases^26^. Generally, it is known that the functional status of female patients with cancer affects their QoL^27–29^. Psychological distress and QoL of female patients with cancer were found to be poorer when functional status was poor, particularly in patients with breast and ovarian cancers^30^.

The association between HGS and HRQoL, as components of functional status in female patients with cancer, can be attributed to various reasons. First, it may be due to physiological differences between men and women. Women generally have relatively lower muscle mass compared to men. This can lead to lower strength and physical function in female patients, which may more clearly manifest in the association with HRQoL^31^. In addition, it is generally acknowledged that women and men experience different types of cancer. For example, breast cancer predominantly occurs in women, while prostate cancer is more common in men. These different types of cancer occur in distinct areas, potentially differently impacting muscle and physical function. Moreover, social roles and environments can induce different experiences between women and men. These experiences can influence the psychological and social aspects of HRQoL after a cancer diagnosis. Women often take on the primary caregiver role in families or may face other societal pressures. Results based on sex- and age-standardized data also indicated lower HRQoL in women compared to men^28^. It was noted that, for female patients with cancer who need to take on the crucial caregiver role in their families, the QoL decreases during active treatment^32^. Moreover, female patients with cancer often face more functional and psychological challenges in their daily lives, especially when lacking family support for self-care during the treatment process.

Lower HGS can imply decreased overall muscle strength and reduced physical endurance, which could be interpreted as indicative of compromised health status^33^. Generally, maintaining an appropriate level of muscle strength is necessary to sustain daily activities and prevent diseases. However, as age advances and disease onset and aging occurs, muscle strength tends to decline^34^. The decrease in muscle mass can impair the body’s balance maintenance and flexibility, leading to direct negative effects on disease prevention and health maintenance. This in turn may cause injuries during activities among patients with cancer^35^. Muscle strength deficiency among patients with cancer poses a significant risk of chronic problems and makes recovery of lost independence particularly challenging. This often results in issues with daily life, physical capabilities, and a heightened likelihood of mental problems^36^. Therefore, the decline in HGS can significantly impact HRQoL in patients with cancer.

Regarding HRQoL, both male and female patients were affected by age, subjective health perception, and stress recognition. This signifies that as age increases, it not only influences physical health but also plays a significant role in the mental well-being of patients with cancer, thereby impacting their HRQoL. When stress recognition is high, a lower HRQoL is observed. Considering the research results that 45% of patients with cancer meet the criteria for post-traumatic stress disorder after trauma^37^, it indicates that patients with cancer experience severe levels of stress. Furthermore, even after the completion of cancer treatment, patients continue to experience psychological distress and stress related to the prognosis and recurrence of cancer^38^. This leads to issues like anxiety and depression, which significantly affect HRQoL^39^. Since cancer is a chronic and long-term disease, interventions addressing stress require more systematic, long-term approaches.

Significant differences were observed in the association of HRQoL with household income and marital status in men. The association between lower income levels and reduced QoL implies that patients with cancer who face substantial economic burdens during treatment may continue to experience economic challenges affecting their QoL even after recovery^40^. Furthermore, the lower HRQoL among unmarried individuals may be attributed to the absence of a support system, making it difficult to detect health problems early and adapt to changes in health status. Among women, those with a middle school education showed significantly lower HRQoL, but there was no notable trend observed for those with higher levels of education.

The strength of this study lies in its use of nationally representative data, which enhances the reliability and validity of the research findings. The use of relative HGS further bolstered the reliability of the results. We also conducted separate analyses for males and females to examine how the relationship between relative HGS and HRQoL differs by sex. This allowed us to gain a clearer understanding of the impact of sex differences on the results. Despite these strengths, this study had several limitations. First, the study employed a cross-sectional analysis, which implies limitations in establishing causality. Therefore, the focus was primarily on assessing associations rather than causal relationships. Second, the KNHANES database does not include information on the date of cancer diagnosis. Additionally, the study did not account for whether participants were currently undergoing treatment or had completed treatment. Therefore, it was difficult to adequately control for the potential impact of patients' functional status and the time lag from cancer diagnosis on current HRQoL and survey responses. Third, given that cancer diagnosis status is identified only via a self-questionnaire and the data is specific to South Korea, prudence is advised when attempting to generalize the findings. Fourth, there may be additional confounding variables that were not considered in the analysis, and these variables have the potential to impact the research results.

## Conclusion

This study analyzed the relationship between HGS and HRQoL among Korean patients with cancer by integrating data from 2014 to 2019. The results showed that utilizing the HGS measurement can provide an opportunity to detect changes in physical function and HRQoL in female patients with cancer, allowing for early intervention or improvement. This can offer crucial information for clinical decision-making and contribute to enhancing HRQoL for patients. Considering these advantages, the HGS measurement can be effectively utilized as a pivotal tool for evaluating HRQoL in female patients with cancer. Further research is necessary to gain a deeper understanding of the difference between HGS and HRQoL in these patients.

## Data Availability

The data that support the findings of this study are available in Korea National Health and Nutrition Examination Survey at https://knhanes.kdca.go.kr. These data were derived from the following resources available in the public domain: Korea National Health and Nutrition Examination Survey, https://knhanes.kdca.go.kr/knhanes/eng/index.do.
